# Supplementary material for: Seminoma and Embryonal Carcinoma Footprints Identified by Analysis of Integrated Genome-Wide Epigenetic and Expression Profiles of Germ Cell Cancer Cell Lines
Source: PLoS One. 2014 Jun 2;9(6):e98330. doi: 10.1371/journal.pone.0098330 (PMC4041891; doi:10.1371/journal.pone.0098330)
Supplement: File S1 — ZIP file containing DMRforPairs output for significant regions. Please start from the html files. (ZIP) [file pone.0098330.s008.zip › figures/38292.pdf]

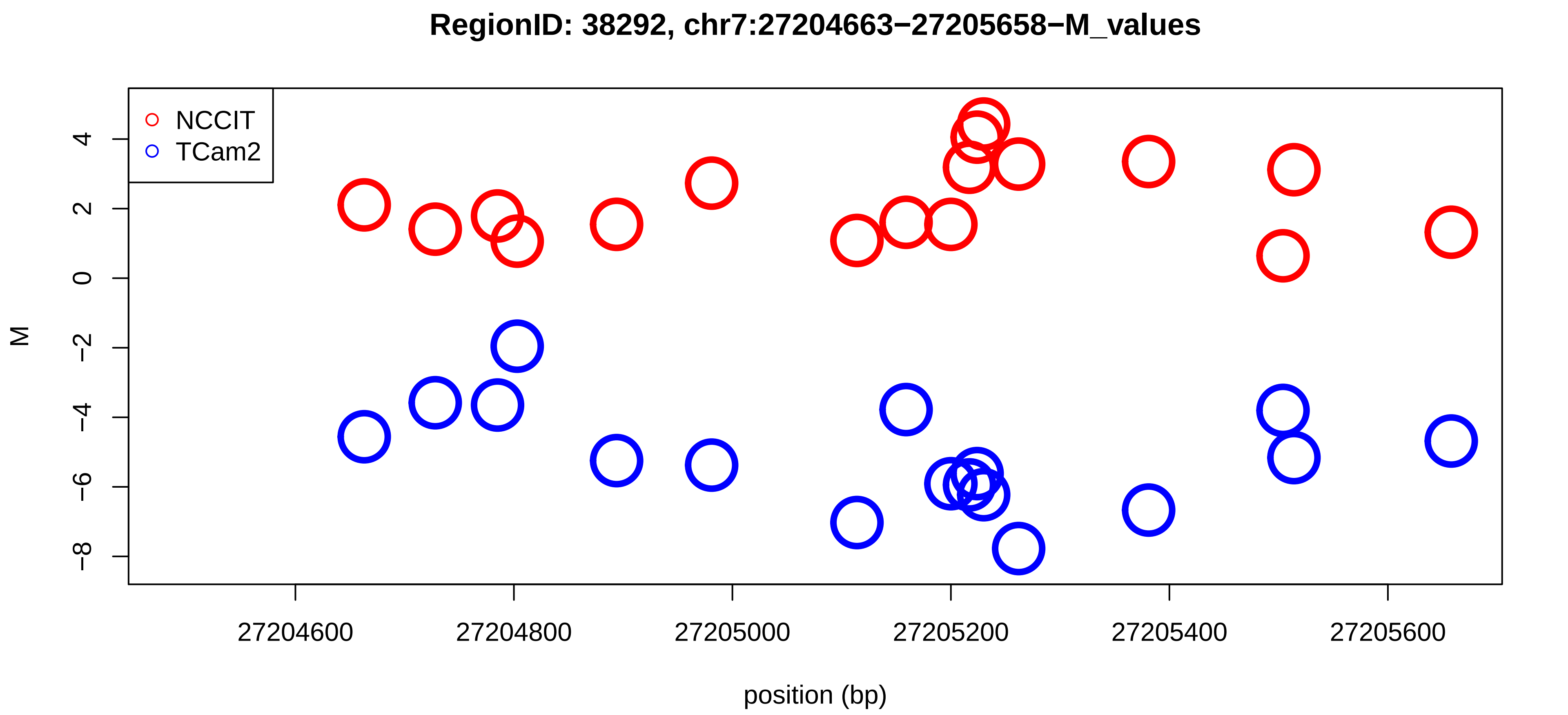

RegionID: 38292, chr7:27204663−27205658−M_values
NCCIT
TCam2
M
4
2
0
−2
−4
−6
−8
27204600
27204800
27205000
27205200
27205400
27205600
position (bp)

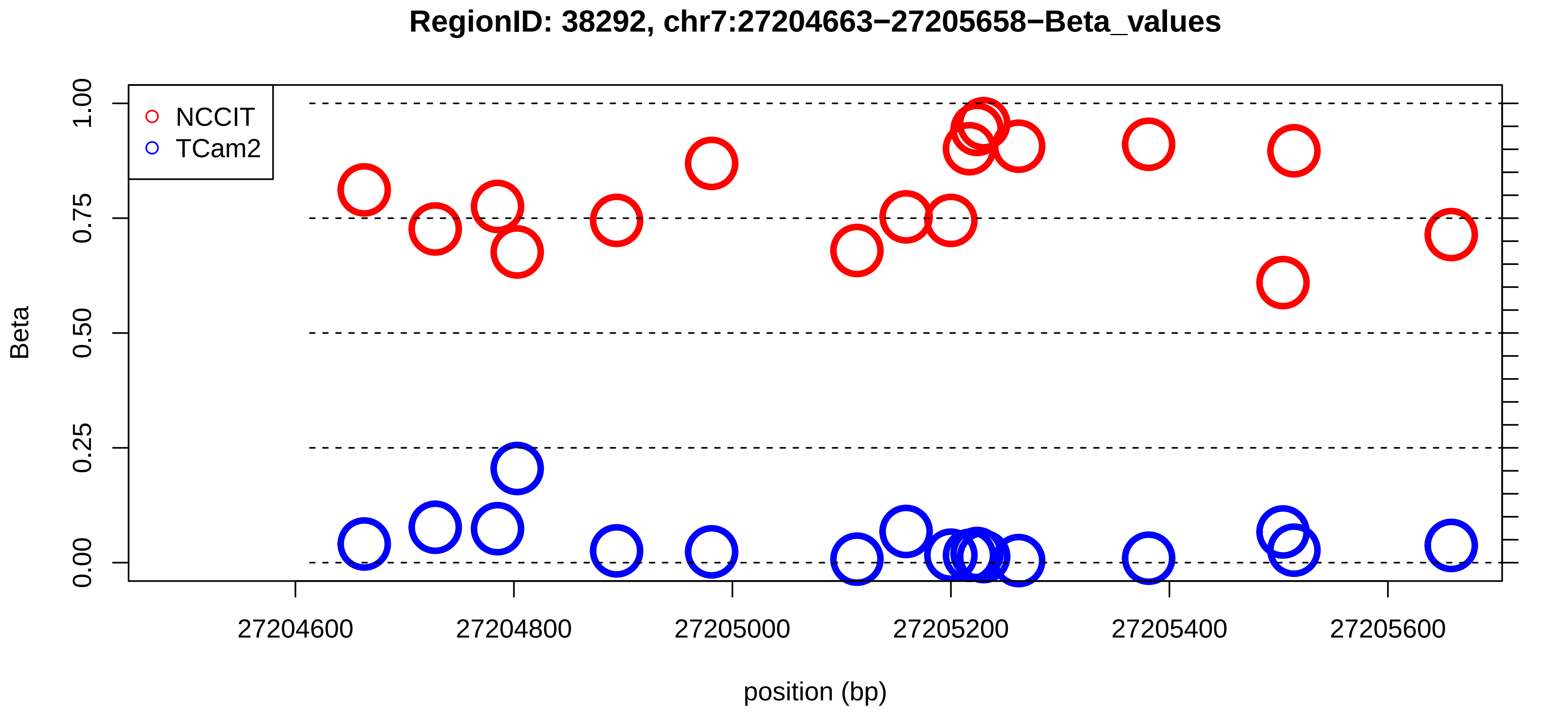

RegionID: 38292, chr7:27204663−27205658−Beta_values
NCCIT
TCam2
Beta
1.00
0.75
0.50
0.25
0.00
27204600
27204800
27205000
27205200
27205400
27205600
position (bp)

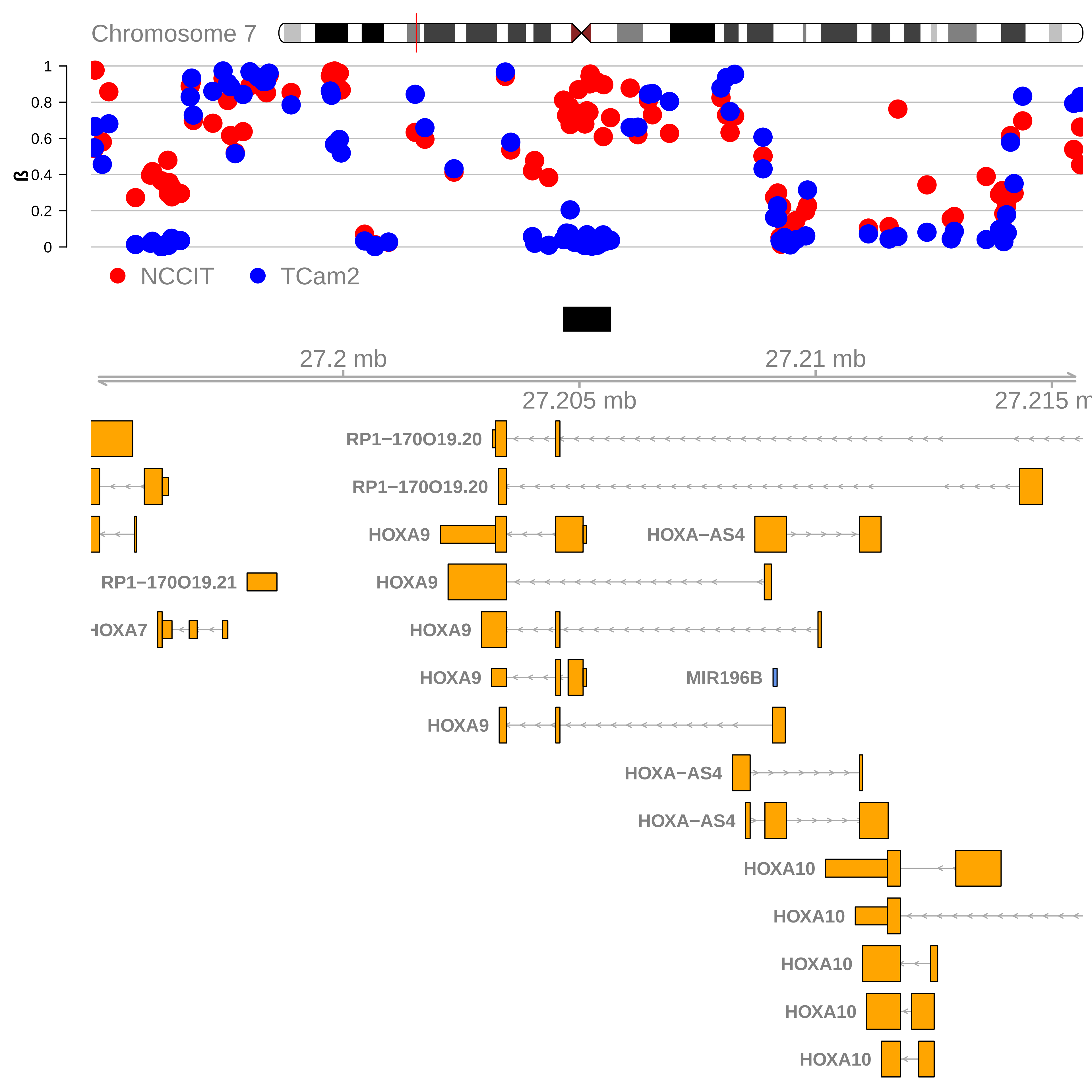

Chromosome 7
1
0.8
0.6
ß 0.4
0.2
0
NCCIT
TCam2
27.2 mb
27.21 mb
27.205 mb
27.215 m
RP1−170O19.20
RP1−170O19.20
HOXA9
HOXA−AS4
RP1−170O19.21
HOXA9
HOXA7
HOXA9
HOXA9
MIR196B
HOXA9
HOXA−AS4
HOXA−AS4
HOXA10
HOXA10
HOXA10
HOXA10
HOXA10
